# Cardiorenal Syndrome: Pathophysiology, Management, and the Challenge of Therapeutic Inertia

**DOI:** 10.3390/medicina62101855

**Published:** 2026-09-25

**Authors:** Guido Gembillo, Vincenzo Calabrese, Luca Soraci, Lorenzo Lo Cicero, Manuel Scapellato, Rossella Messina, Maria Federica Ricca, Maria Rita Stancanelli, Andrea Corsonello, Domenico Santoro

**Affiliations:** 1 Unit of Nephrology and Dialysis, Department of Clinical and Experimental Medicine, University of Messina, 98125 Messina, Italy; manuelscapellato@gmail.com (M.S.); rossy.messina92@gmail.com (R.M.); mariafedericaricca8@gmail.com (M.F.R.); dsantoro@unime.it (D.S.); 2 Department of Medicine and Surgery, “Kore” University of Enna, 94100 Enna, Italy; v.calabrese@outlook.it (V.C.); mariarita.stancanelli@gmail.com (M.R.S.); 3 Unit of Geriatric Medicine, Italian National Research Center on Aging (IRCCS INRCA), 87100 Cosenza, Italy; l.soraci@inrca.it (L.S.); a.corsonello@inrca.it (A.C.); 4 PhD Program in Molecular and Clinical Medicine, Department of Health Promotion, Mother and Child Care, Internal Medicine and Medical Specialties “G. D’Alessandro” (PROMISE), University of Palermo, 90127 Palermo, Italy; lorenzolocicero95@gmail.com; 5 Department of Pharmacy and Health and Nutritional Sciences, University of Calabria, 87036 Rende, Italy

**Keywords:** cardiorenal syndrome, chronic kidney disease, heart failure, therapeutic inertia, guideline-directed medical therapy, SGLT2 inhibitors, finerenone, semaglutide, implementation science

## Abstract

Cardiorenal syndrome describes the clinical consequences of the bidirectional influence that cardiac and renal dysfunction exert on each other—a domain in which single-organ care proves insufficient. A 2024 meta-analysis estimated the pooled prevalence of chronic kidney disease among patients with heart failure at 49%. Across individual cohorts, this figure ranges between 30% and 60%, with the spread reflecting the population studied and the threshold used to define kidney disease. Among acute heart failure admissions, 64% of cases have an estimated glomerular filtration rate below 60 mL/min/1.73 m^2^, and mortality reaches 7.6% once it falls into the severe range (15–29 mL/min/1.73 m^2^). The same can be described in the other “direction”: congestive heart failure is reported in 28% of adults aged 66 years or older with chronic kidney disease, compared to 6% of those without. The Acute Dialysis Quality Initiative distinguishes a five-subtype framework by direction and chronicity of organ injury, and each subtype shows characteristic prescribing failure patterns. In types 1 and 2, the dominant problem is premature de-escalation of neurohormonal therapy at the first creatinine rise. In type 4, the failure is upstream in the cascade: cardiovascular surveillance lags behind the increasing rate of chronic kidney disease. Randomized trials over the past decade have expanded the therapeutic options. Sodium-glucose cotransporter 2 inhibitors showed cardiorenal benefit across every ejection fraction studied and at estimated glomerular filtration rate values down to 20 mL/min/1.73 m^2^. These findings have expanded treatment eligibility to patients with advanced CKD. The FINE-HEART pooled analysis of finerenone suggested a nominal reduction in all-cause mortality, alongside fewer heart failure hospitalizations and improvement in the kidney composite outcome. In the FLOW trial, semaglutide showed a reduction in the composite of major kidney events and cardiovascular death in diabetic adults with chronic kidney disease. In this cohort, the estimated glomerular filtration rate slope was slower in the active arm and secondary outcomes showed reductions in major adverse cardiovascular events and in all-cause mortality. However, observational registries show that these classes of drugs are less used in patients with the highest baseline risk, with advancing renal disease identified as an independent predictor of under-prescription. Therapeutic inertia in this setting reflects determinants at the patient, clinician, and system levels, which is compounded because each prescribing decision must be justified for both the heart and the kidney. The primary objective of the present narrative review is the therapeutic inertia that separates this evidence from routine prescribing: the sections on pathophysiology, imaging and biomarker tools, and subtype-specific pharmacotherapy supply the clinical rationale, and the closing sections cover the implementation strategies and the multidisciplinary framework required to act on the trial findings.

## 1. Introduction

Heart failure (HF) and chronic kidney disease (CKD) frequently coexist in older adults, where their bidirectional interaction defines a distinct clinical phenotype associated with worse outcomes than either condition alone. The two conditions overlap substantially [1,2]. Pooled estimates from studies of heart failure populations put the prevalence of CKD at 49% (53% in acute and 42% in chronic presentations) [3], which serves as the best available point estimate; across individual cohorts, this figure ranges between 30% and 60%, and the width of that range reflects the population studied and the threshold used to define kidney disease more than any real difference in disease burden. The Acute Decompensated Heart Failure National Registry (ADHERE) included 118,465 hospitalizations from multiple centers across the United States (US). Among admissions, the estimated glomerular filtration rate (eGFR) was 90 mL/min/1.73 m^2^ or higher in 10,660 patients (9.0%), mildly reduced (60–89) in 32,423 patients (27.4%), moderately reduced (30–59) in 51,553 patients (43.5%), severely reduced (15–29) in 15,553 patients (13.1%), while 8276 patients (7.0%) were either in kidney failure or on chronic dialysis. About 63.6% of the whole cohort had an eGFR below 60 mL/min/1.73 m^2^ at admission. The in-hospital mortality increased sharply with decreasing kidney function: from 1.9% for eGFR ≥ 90 mL/min/1.73 m^2^ to 7.6% between 15 and 29 mL/min/1.73 m^2^, and 6.5% for patients in kidney failure or requiring dialysis [4]. Mortality in the kidney failure stratum was therefore lower than in the eGFR 15–29 stratum, an inversion that calls for comment. Several explanations are plausible: patients established on maintenance dialysis have survived the transition to renal replacement therapy, thereby removing the highest-risk individuals from the stratum; volume can be removed by ultrafiltration once diuretic responsiveness is lost, making decongestion achievable where it is difficult at an eGFR of 15–29 mL/min/1.73 m^2^; and the stratum pools patients on chronic dialysis with those in undialyzed kidney failure, whose risks differ. The registry analysis was not designed to separate these effects, and the pattern should be interpreted as phenomenon driven by competing risk and treatment intensity [4].

In the Atherosclerosis Risk in Communities (ARIC) cohort, including 14,857 adults free of HF at baseline, with a mean follow-up of 13.2 years, the crude incidence of HF was threefold higher in those entering the study with an eGFR less than 60 mL/min/1.73 m^2^ than in those with preserved renal function. In multivariate cox regression models, the risk for incident HF was 94% higher than in those with preserved renal function (HR, 95% CI: 1.94, 1.49–2.53) [5]. In the United States Renal Data System, 28% of older adults with CKD had congestive HF, compared to only 6% of those without CKD [6]. The Kaiser Permanente cohort of 1,120,295 adult outpatients quantified the risk gradient across eGFR strata: with the eGFR ≥ 60 mL/min/1.73 m^2^ stratum as a reference, the adjusted hazard ratios for all-cause mortality were 1.2, 1.8, 3.2, and 5.9 across eGFR strata of 45–59, 30–44, 15–29, and below <15 mL/min/1.73 m^2^, respectively; the corresponding ratios for cardiovascular events were 1.4, 2.0, 2.8, and 3.4 [7].

The Chronic Renal Insufficiency Cohort (CRIC) followed 3791 adults with mild-to-severe CKD (eGFR 20–70 mL/min/1.73 m^2^) and reported adjusted rate ratios for hospitalizations due to heart failure of 2.2 when the baseline eGFR was less than 30 mL/min/1.73 m^2^ and 2.6 when the urine albumin-to-creatinine ratio (UACR) was 300 mg/g or above. In this cohort, 20.6% of CRIC participants admitted for HF were readmitted within 30 days of discharge. The subsequent renal trajectory was affected by HF hospitalizations. Among patients with eight or more HF hospital days during the first two years of follow-up, summed across admissions, the adjusted hazard ratio was 1.90 (95% CI 1.15–3.15) for CKD progression and 2.91 (95% CI 2.17–3.90) for death [8]. The 2019 Kidney Disease: Improving Global Outcomes (KDIGO) Controversies Conference on HF in CKD outlined the shared pathophysiology and the structural failures of usual care [9]. From a renal perspective, the 2024 KDIGO CKD guideline re-endorsed sodium-glucose cotransporter 2 (SGLT2) inhibitors, renin–angiotensin–aldosterone system (RAAS) inhibitors, and statins as standard pharmacotherapy, adding non-steroidal mineralocorticoid receptor antagonists where appropriate; underutilization of these agents is associated with higher rates of cardiorenal events [10].

The Acute Dialysis Quality Initiative (ADQI) framework, initially proposed in 2008 and further expanded upon in the 2019 American Heart Association (AHA) scientific statement [11,12,13], categorized five subtypes based on initial organ failure and time course. Registry data make clear the clinical cost of neglecting it. In CHAMP-HF, a registry of 3518 US outpatients with HF with reduced ejection fraction (HFrEF), 27% were not prescribed any ACE inhibitor (ACEi), angiotensin receptor blocker (ARB), or angiotensin receptor–neprilysin inhibitor (ARNi). Additionally, 33% of regimens were missing β-blockers, and the omission of mineralocorticoid receptor antagonists (MRAs) was as high as 67%. Absolute contraindications were recorded in less than 2% of eligible patients. Renal insufficiency was an independent predictor of prescribing omissions and under-prescription, and the achieved doses were also lower at each step, with only 1% of eligible patients reaching target doses in all three classes [14]. The gap between published evidence and bedside practice appears to reflect, at least in part, limitations in treatment delivery. This gap meets the definition of therapeutic inertia introduced by Phillips and colleagues, which was broadened to clinical inertia by Khunti and Davies [15,16]; this framework is detailed in Section 8.1.

The primary objective of this narrative review is to examine therapeutic inertia in cardiorenal syndrome (CRS), including why treatments with proven cardiorenal benefit reach a minority of eligible patients, at which level of care the delay arises for each CRS subtype, and which countermeasures have evidence behind them. The sections on pathophysiology, diagnostic tools, and pharmacotherapy supply the clinical rationale for that argument, and the link to inertia is made explicit at the close of each of section. The following sections address subtype-specific therapy and pharmacological evidence of cardiorenal protection, with a focus on the role of glucagon-like peptide-1 receptor agonists. The three-level taxonomy of therapeutic inertia adapted from diabetic kidney disease (DKD) [17] is presented next. The multidisciplinary care framework and economic rationale for earlier action are described in the final sections.

Given the narrative design, the literature search was structured without being systematic. PubMed/MEDLINE, Embase, Scopus, and Web of Science were searched from inception to 1 June 2026 using the terms cardiorenal syndrome, heart failure, chronic kidney disease, worsening renal function, therapeutic inertia, clinical inertia, guideline-directed medical therapy, SGLT2 inhibitor, finerenone, GLP-1 receptor agonist, potassium binder, frailty, and implementation, alone and in combination. Reference lists of guidelines, consensus documents, and reviews were also screened. Randomized trials and their prespecified or post hoc analyses, meta-analyses, registry, and cohort studies with quantitative data on prescribing gaps, and current KDIGO, ESC/HFA, and AHA documents were given priority. Conference abstracts, case reports, and papers in languages other than English were excluded. In the text, the design of each study is stated where it affects the weight of a claim; specifically, observational findings are described as associations, subgroup and post hoc results as suggestive, and statements based on clinical experience as commentary.

## 2. Hemodynamics, Venous Congestion, and the Failure of the Forward-Flow Model

Changes in blood flow perfusion represent the most studied cause of worsening renal function in patients with acute decompensated HF. However, the model is incomplete and often misleading. Mullens and colleagues studied 145 consecutive patients followed with pulmonary artery catheter monitoring. Those who develop deteriorating renal function had a higher mean central venous pressure (CVP) on admission (18 ± 7 mmHg) compared to those who did not (12 ± 6 mmHg, *p* < 0.001). The CVP correlation remained even after correction for hemodynamic and renal variables such as the cardiac index, pulmonary capillary wedge pressure, and admission eGFR [18]. The data were from a single tertiary-center series and no formal testing of wider generalizability was undertaken in larger multicenter cohorts. This mechanism is a leading physiological explanation for the syndrome: renal venous pressure is transmitted into the non-distensible renal capsule, raising interstitial hydrostatic pressure above the perfusion gradient. Ross et al. reported that congestion could trigger a functional kidney compartment syndrome [19]. Compression can be compounded by intra-abdominal hypertension arising from ascites and visceral edema. These mechanisms trigger tubular hypoxia. Galectin-3 and kidney injury molecule-1 (KIM-1) have been proposed as mediators of the downstream injury pathways on the basis of association and expression studies, and their causal contribution in human cardiorenal syndrome has yet to be demonstrated [20,21]. The venous congestion mechanism is often missed, and even mild creatinine elevations can be misinterpreted. The downstream cost in terms of compromised decongestion and suboptimal guideline-directed medical therapy (GDMT) dosing has been placed at the center of the problem in the Heart Failure Association (HFA) position statement about evaluating kidney function [22]. If integrated clinical and imaging data suggest congestion as the dominant driver, stopping the diuretic at the first rise in serum creatinine may worsen the outcome. If there is frank hypovolemia or pre-renal physiology, the reverse is true. Point-of-care bedside imaging now offers a feasible strategy for directing decongestion in cardiorenal patients. Ultrasound of the inferior vena cava provides an estimate of right-side filling pressures, and intra-renal venous Doppler may show the characteristic discontinuous diastolic pattern of renal venous hypertension. Even where imaging is unavailable, formally measuring jugular venous pressure provides valuable data [11,18,22]. Spot urinary sodium measured two hours after intravenous furosemide provides further useful data. This analysis is a direct measure of natriuresis and is more actionable than a static creatinine value and less dependent on baseline renal function [23]. The PUSH-AHF trial randomized 310 patients with acute HF to natriuresis-guided therapy or standard of care. In the intervention arm, spot urinary sodium was measured at 2 and 6 h, and again at set time points up to 36 h. Values below 70 mmol/L triggered treatment intensification. The early spot sample described by Testani et al. served as the bedside decision tool, while the 24 h urinary sodium served as a co-primary endpoint summarizing the response over the first day. The two measurements therefore complemented each other, and neither superseded the other. The 24 h urinary sodium reached 409 mmol compared to 345 mmol in the standard-care arm (*p* = 0.0061). Treatment was intensified in 85% patients within 36 h in natriuresis-guided therapy, without a significant excess of adverse renal or electrolyte events. The composite of all-cause mortality and rehospitalization for HF at 180 days was similar between groups (HR 0.92, 95% CI 0.62–1.38). An exploratory analysis noted a numerical trend toward improved in-hospital mortality favoring the intervention, with two deaths in the natriuresis-guided arm compared to fourteen in the standard-care arm [24]. The Venous Excess Ultrasound (VExUS) score is a combination of hepatic, portal, and intrarenal venous Doppler with inferior vena cava plethora; higher VExUS grades are associated with an increased risk of acute kidney injury and identify residual venous congestion that might be missed during physical examination [25]. The B lines on lung ultrasound are a quantitative index of pulmonary congestion that change with treatment within hours, often before any improvement in symptoms or creatinine [26]. The combination of modalities could help characterize residual congestion and may assist in identifying patients that are decongested but not yet ready for discharge [27]. These modalities complement clinical and biomarker assessment, which remains the basis of the evaluation.

Decompensation involves hemodynamic disturbance together with neurohormonal activation, triggered by stimulation of the RAAS, adrenergic renal vasoconstriction, and vasopressin-mediated water retention, with subsequent hyponatremia. Failure to obtain a significant reduction (<30% from baseline) in NT-pro-BNP before discharge is independently associated with early rehospitalization and cardiovascular death [28]. Aldosterone-induced myocardial and renal fibrogenesis is largely hemodynamically independent and supports the long-term use of MRAs after the acute decompensation has been treated and possibly resolved [29]. For the inertia argument, the relevance of this section lies in interpreting the first creatinine rise. When congestion is misinterpreted as hypoperfusion, decongestion and GDMT are both withheld, causing the delay to begin at the bedside assessment, before any prescribing decision is taken.

## 3. Molecular Mediators of Irreversible Injury

The transition from hemodynamic dysfunction to fibrotic scarring is caused by the convergence of biological pathways activated by the initial cardiorenal insult, which are reactivated with each subsequent episode of decompensation. In a pooled analysis of the Clinical Phenotyping and Resource Biobank (C-PROBE) study and the Seattle Kidney Study, a doubling of circulating galectin-3 was associated with a 35% higher adjusted risk of progression to an eGFR below 15 mL/min/1.73 m^2^ or end-stage renal disease (aHR 1.35, 95% CI 1.01–1.80), independently of baseline kidney function and comorbidities [20]. Soluble ST2 (sST2) is a circulating decoy receptor for interleukin-33 (IL-33) that rises in response to ventricular wall stress and is thought to attenuate an anti-fibrotic, cardioprotective signal in target tissues. By contrast, in the Cardiovascular Health Study (2763 older adults free of HF at baseline), neither galectin-3 nor sST2 was independently associated with subsequent kidney function decline [30]. The underlying biology of both markers converges on myocardial strain and renal fibrogenesis, but neither marker has yet been validated as a therapeutic target in adequately powered intervention trials.

KIM-1 is expressed on the apical surface of proximal tubular cells after injury, enabling the surviving tubular epithelium to ingest apoptotic debris and prevent intraluminal obstruction. Sustained KIM-1 expression under chronic or recurrent stress has been associated with macrophage recruitment and tubular uptake of filtered proteins, sustaining a pro-inflammatory loop in experimental models, but human data in CRS are limited [21]. In a prospective cohort of 146 patients admitted with acute decompensated HF investigated for CRS type 1 prediction, Atici et al. found a moderate negative correlation between the percentage change in urinary KIM-1 and the percentage change in creatinine after diuretic initiation (r = −0.357, *p* = 0.016). A decrease in KIM-1 in patients developing acute kidney injury was interpreted as dynamic tubular adaptation, which limits the use of KIM-1 as a static severity marker, and KIM-1 did not independently predict the development of type 1 CRS [31]. None of these markers has reached a stage at which they could guide a prescribing decision, so the inertia described in Section 8 currently operates on creatinine and eGFR alone.

## 4. Subtype-Specific Management Across the CRS Spectrum

The five subtypes of CRS in Table 1 vary in pathophysiology, prognosis, and decision logic; the therapeutic priorities identified here are consistent with this taxonomy.

Type 1 CRS is caused by acute decompensated HF, which triggers acute kidney injury (AKI). A key management question is whether the rise in serum creatinine levels is a marker of poor renal perfusion or poor decongestion. Large volume overload is characterized by venous congestion and incomplete decongestion [18]. Loop diuretics remain first-line, although diuretic resistance is common and requires combination strategies that block sodium reabsorption at other sites in the nephron. The ADVOR trial showed this principle with acetazolamide. Investigators randomized 519 patients admitted with acute decompensated HF and clinical volume overload to intravenous acetazolamide (500 mg once daily) or placebo, with both arms receiving standard intravenous loop diuretics at twice the oral maintenance dose. At 72 h, successful decongestion was achieved in 42.2% of patients receiving acetazolamide compared to 30.5% receiving placebo (relative risk 1.46, 95% CI 1.17–1.82, *p* < 0.001). Length of stay was also shorter, and a higher proportion of patients left hospital free of congestion [32]. Acetazolamide inhibits bicarbonate and consequent sodium reabsorption in the proximal tubule, a complementary action to that of SGLT2 inhibitors, although the concomitant use of SGLT2 inhibitors was excluded by design in the ADVOR study. The CLOROTIC trial randomized 230 patients who were receiving oral furosemide (or equivalent) at a dose of at least 80 mg for at least one month before admission. Hydrochlorothiazide plus intravenous furosemide was significantly more effective than placebo for 72 h weight loss. In the hydrochlorothiazide arm, deterioration of renal function was more frequent, without an increase in mortality or rehospitalization, and the benefit of weight loss was reduced at an eGFR below 45 mL/min/1.73 m^2^ [33]. The EMPULSE trial demonstrated the benefits of SGLT2 inhibition in 530 stabilized patients hospitalized for acute HF. Starting empagliflozin during the hospital stay was associated with a significant composite clinical benefit at 90 days (win ratio 1.36, 95% CI 1.09–1.68, *p* = 0.0054), and this benefit was consistent across subgroups of renal function down to an eGFR of 20 mL/min/1.73 m^2^. Acute renal failure and any early decline in eGFR resolved by day 90 were less common in the active arm (7.7% vs. 12.1%) [34,35].

Another possibility is ultrafiltration, which may be considered when decongestion based on diuretic use fails due to natriuretic resistance or hemodynamic intolerance, or when worsening renal function limits the ability to escalate dose. The CARRESS-HF trial randomized 188 patients with acute decompensated HF, worsening renal function, and persistent congestion to ultrafiltration or stepped pharmacotherapy. The pharmacological approach had better renal outcomes at 96 h, similar weight loss, and fewer serious adverse events [36]. The trial dates from 2012 and enrolled 188 patients, so it carries the weight of a single, modestly sized study and deserves the same caution applied to the single-center congestion data in Section 2. Ultrafiltration should not replace optimized diuretic therapy, and its role once pharmacological options are exhausted remains unclear from randomized data. In type 1 CRS with AKI stages 2 or 3, continuous renal replacement therapy is generally indicated for metabolic reasons, and its effect on cardiac recovery and longer-term renal outcomes is supported by observational data only [37].

Type 2 CRS is a progressive CKD secondary to chronic cardiac dysfunction. Type 2 is the CRS subtype most studied by HF and CKD trials. The PARADIGM-HF trial demonstrated a reduction in cardiovascular death or hospitalization for HF with sacubitril/valsartan compared to enalapril (HR 0.80, 95% CI 0.73–0.87, *p* < 0.001). This benefit was seen in all KDIGO risk categories of kidney disease [38,39]. In a UACR substudy of 1910 participants of the PARADIGM-HF trial, 26% were considered high or very high risk [40]. Chatur et al. conducted parallel analyses of the PARADIGM-HF and PARAGON-HF trials and reported that a moderate reduction in eGFR (>15%) during sacubitril/valsartan therapy was not consistently associated with adverse outcomes and did not influence long-term benefits in either trial [41]. These data support reconsidering routine discontinuation of ARNi after an initial rise in creatinine. The DAPA-CKD trial demonstrated a 39% risk reduction for sustained ≥50% decline in eGFR, progression to end-stage kidney disease, or renal/cardiovascular death (HR 0.61, 95% CI 0.51–0.72, *p* < 0.001), in both diabetic and non-diabetic CKD and across eGFR strata, including patients with an eGFR as low as 25 mL/min/1.73 m^2^ [42]. Similarly, the EMPA-KIDNEY trial extended this evidence to a broader CKD population, enrolling participants down to an eGFR of 20 mL/min/1.73 m^2^ and, unlike the DAPA-CKD trial, without requiring albuminuria; empagliflozin reduced kidney disease progression or cardiovascular death (HR 0.72, 95% CI 0.64–0.82, *p* < 0.001) [43]. The early eGFR decline with SGLT2 inhibitors is thought to be due to the reversal of glomerular hyperfiltration, which is the mechanism driving their nephroprotective effect. The HFA consensus document emphasizes that a transient decrease in eGFR is expected after initiation of RAAS inhibition, ARNi, MRA, or SGLT2 inhibitors; this decline is usually clinically benign and does not warrant discontinuation or down-titration per se [44]. Damman et al. performed a meta-analysis of 28 studies that included 49,890 patients with HF and showed that worsening renal function was independently associated with increased mortality (adjusted HR 1.95, 95% CI 1.45–2.62) [45]. The two statements refer to different situations: the HFA consensus describes the modest, early, hemodynamically mediated fall in eGFR that follows initiation or up-titration of a disease-modifying drug in a stable patient, whereas the meta-analysis pooled worsening renal function of any cause, much of it occurring during hospitalization for decompensation, where a creatinine rise more often reflects persisting congestion, hypoperfusion, or true tubular injury. The HFA consensus underlines that the prognostic relevance of these findings is highly dependent on the clinical context, especially the degree of decongestion at the time of the switch [44]. Renal function after mechanical circulatory support illustrates the same cardiac-to-kidney dependence over a longer horizon. Brisco et al. analyzed 3363 adults enrolled in the Interagency Registry for Mechanically Assisted Circulatory Support (INTERMACS) trial. Early post-mechanical circulatory support (MCS) recipients had a median 48.9% improvement in eGFR, but this benefit was largely transient, and the one-year mean eGFR was only 6.7% above the pre-MCS baseline. Increased all-cause mortality was associated with both initial improvement (adjusted HR 1.64, 95% CI 1.19–2.26, *p* = 0.002) and further deterioration (adjusted HR 1.63, 95% CI 1.15–2.13, *p* = 0.004). The U-shaped pattern indicates that even a major hemodynamic intervention does not yield a single directional index of cardiorenal recovery [46].

Type 3 CRS, where AKI leads to acute cardiac dysfunction, is generally regarded as a cardiac complication of AKI, and the label of a distinct CRS subtype is applied less often. The usual precipitants are the common causes of hospital-acquired AKI: contrast-associated AKI, sepsis-associated AKI, rhabdomyolysis, cardiac surgery and acute glomerulonephritis [11,12]. The cardiac consequences listed in Table 1 arise through several mechanisms that act within hours to days. Sodium and water retention produces volume overload and pulmonary edema, while hyperkalemia and metabolic acidosis alter myocardial excitability and contractility, accounting for arrhythmia and conduction disturbances. Furthermore, retained uremic solutes contribute to pericarditis and myocardial depression, and the neurohormonal, sympathetic, and inflammatory activation that accompanies AKI adds further myocardial insult. Consequently, an episode of AKI can precipitate decompensation in a heart with limited reserve [11]. Monitoring rests on serial electrolytes, electrocardiography, and echocardiography whenever volume status or conduction changes because biomarker guidance remains weak in this setting. Troponin and natriuretic peptides both accumulate as glomerular filtration falls, thresholds validated in patients with preserved kidney function lose specificity, and cut-offs specific to AKI have yet to be validated [11,22]. The timing of cardiac surveillance depends on the conceptualization of the syndrome. The relative lack of evidence from prospective trials in type 3 CRS compared to other subtypes was noted in the 2019 American Heart Association scientific statement and reflects the historical separation of cardiac and renal endpoints in trial design [11]. Control of volume is especially difficult when there is a pre-existing cardiac dysfunction that limits the safety margin between adequate resuscitation and progressive congestion.

Type 4 CRS is the most prevalent subtype at the population level, given the high cardiovascular burden in CKD patients. This population needs systematic cardiovascular risk stratification, which has not yet been incorporated into routine CKD care. What the right content and frequency of such surveillance should be is an open question for guideline-directed work. The KDIGO 2024 CKD guideline emphasized the importance of more intensive cardiovascular assessment and risk stratification in CKD patients at high risk of HF [10]. The KDIGO 2026 Heart Failure and CKD Conference report further refined the surveillance framework: CKD and albuminuria should be incorporated into the diagnostic pathways of heart failure, and kidney function trajectories should be more closely followed on GDMT [2,9]. In addition to pharmacotherapy, device-based interventions might be considered on an individual basis in the CKD–HF population. In HFrEF patients with a wide QRS, cardiac resynchronization therapy was associated with lower morbidity and mortality in the CARE-HF trial, with a 37% relative risk reduction in death from any cause or unplanned cardiovascular hospitalization (HR 0.63, 95% CI 0.51–0.77, *p* < 0.001) [47]. However, the trial included only a few patients with an eGFR below 60 mL/min/1.73 m^2^, and extrapolation to advanced CKD rests on a consistent direction of effect, in the absence of direct evidence. In a patient-level meta-analysis of the MADIT-I, MADIT-II and SCD-HeFT trials, the benefit of primary prevention implantable cardioverter-defibrillators decreased with decreasing eGFR. This attenuation reflects a competing risk of non-arrhythmic mortality that overshadows the arrhythmic risk that the device was designed to treat, with the absolute benefit decreasing with worsening renal impairment. Notably, dialysis-dependent patients are poorly represented in this setting [48]. Both trial datasets also predate the ARNi and SGLT2 inhibitor era, so the background therapy against which device benefit was measured differs from current quadruple therapy. Consequently, the incremental benefit of resynchronization or defibrillator therapy in a patient with CKD who already receives contemporary GDMT is uncertain. Therefore, beyond classic ejection fraction and QRS criteria, competing risk profiles should always be considered for selection.

Type 5 CRS is defined as simultaneous cardiac and renal dysfunction driven by a common systemic insult, without the sequential organ failure that defines the other subtypes. This is the classic setting for sepsis. Examples are hepatorenal syndrome in liver cirrhosis and severe autoimmune vasculitis. Therapy targets the systemic driver, followed by management of the single-organ manifestations. Septic CRS is a triad consisting of distributive shock, inflammatory myocardial depression, and ischemic tubular injury. Restoring systemic circulation may, per se, lead to volume overload of a heart with reduced functional reserve. In the setting of portal hypertension and splanchnic pooling in hepatorenal syndrome, a vasodilatory state with effective volume depletion occurs despite whole body fluid overload. Terlipressin improves renal outcomes by increasing renal perfusion pressure through V1-receptor-mediated splanchnic vasoconstriction [11]. In the CONFIRM trial, terlipressin with albumin reversed hepatorenal syndrome more often than placebo (32% versus 17%), but respiratory failure was more frequent on active treatment (14% versus 5%) and 90-day survival did not differ [49]. The respiratory signal shaped the regulatory history of the drug. It received approval by the US Food and Drug Administration only in 2022 and carries a boxed warning for serious or fatal respiratory failure, with volume overload and grade 3 acute-on-chronic liver failure identified as risk factors [50]. In a patient whose cardiac function is already compromised, volume status therefore needs to be assessed before treatment is started, and albumin loading requires the same caution.

## 5. Pharmacological Management of the Cardiorenal Interface

In HFrEF, GDMT comprises ARNi or RAAS inhibitors, β-blockers, MRA, and SGLT2 inhibitors. The evidence base is strong, yet uptake remains poor when CKD coexists. Reviewing the landmark randomized trials across all four classes, Beldhuis and colleagues showed that the reduction in cardiovascular death or heart-failure hospitalization was preserved at every CKD stage for which evidence existed, with no signal of attenuated benefit in higher-risk renal subgroups [51]. Patients with an eGFR below 20 mL/min/1.73 m^2^ and those on maintenance dialysis remain underrepresented in randomized data, so any conclusion in these groups rests on extrapolation. Based on the EMPEROR-Preserved and DELIVER trials, the 2023 European Society of Cardiology (ESC) Focused Update elevated SGLT2 inhibitors to a Class IA recommendation in HFmrEF and HFpEF [52]. The KDIGO 2024 CKD guideline endorsed continuation of GDMT in CKD unless a specific clinical trigger warrants reassessment, applying a 30% threshold for serum creatinine rise within four weeks of initiation of hemodynamically active therapy [10]. Down-titration may be considered when symptomatic hypotension occurs, when an unexplained creatinine rise of more than 30% from a stable baseline persists, or when hyperkalemia proves refractory to management. In each instance, the precipitant should be identified and addressed before the drug itself is reduced [10,44].

Hyperkalemia, real or anticipated, is among the commonest reasons for premature dose reduction or withdrawal of RAAS inhibitors and MRAs when CKD and HF coexist. The pattern is most pronounced when potassium sits in the 5.0–5.5 mmol/L range, where the arrhythmic risk attributable to the electrolyte itself is modest compared to the prognostic cost of losing GDMT. The DIAMOND trial addressed this dilemma directly. Among 1642 patients screened, 1195 with HFrEF and current or prior hyperkalemia-related complication on RAAS blockade entered a single-blind run-in phase on patiromer. During this phase, ACEi, ARB, or ARNi were up-titrated to at least 50% of the target and MRAs were up-titrated to 50 mg daily. Ultimately, 1038 patients completed the run-in phase, and 878 of these (84.6%) reached the specified target doses and were randomized 1:1 (439:439) to continued patiromer or placebo. Continued patiromer reduced the risk of serum potassium exceeding 5.5 mmol/L by 37% (HR 0.63, 95% CI 0.45–0.87, *p* = 0.006), the risk of MRA dose reduction below the target by 38% (HR 0.62, 95% CI 0.45–0.87, *p* = 0.006), and total adjusted hyperkalemia events per 100 patient-years by 34% [53]. Patiromer therefore enables continuation of GDMT by removing the constraint that hyperkalemia would otherwise impose. Sodium zirconium cyclosilicate (SZC), the second binder to enter the market, was tested through a comparable design in REALIZE-K, a randomized withdrawal trial in HFrEF (LVEF ≤ 40%, NYHA II–IV) with prevalent or incident hyperkalemia under structured spironolactone titration. Among 203 participants who attained normokalemia on at least 25 mg of spironolactone during the SZC run-in phase, continuing SZC over six months yielded a significantly higher rate of the primary composite outcome compared to placebo: 71.2% on SZC maintained normokalemia while remaining on spironolactone without rescue therapy across months 1–6, compared to 35.7% on placebo (OR 4.45, 95% CI 2.89–6.86, *p* < 0.001). Four of five hierarchically tested secondary endpoints (normokalemia at randomization dose of spironolactone without rescue, daily spironolactone ≥ 25 mg, time to hyperkalemia, time to spironolactone reduction, or discontinuation for hyperkalemia) likewise favored SZC. The Kansas City Cardiomyopathy Questionnaire (KCCQ) summary score showed no between-group difference at six months (mean treatment difference −1.01, 95% CI −6.64 to 4.63, *p* = 0.72). In an exploratory analysis, the composite of cardiovascular death or worsening HF occurred in 11 participants (11%) on SZC—ten of which were HF events—compared to 3 participants (3%) on placebo, with a nominal log-rank *p*-value of 0.034. The trial was not powered for clinical outcomes, and the investigators presented this finding as a factor to weigh in individual decisions rather than the establishment of harm [54]. A plausible mechanism exists: SZC exchanges potassium for sodium and hydrogen ions, thereby adding a sodium load, and edema is a labeled adverse effect of the compound. By contrast, patiromer exchanges potassium for calcium. In an observational comparison of patients starting either binder, SZC was associated with a higher risk of hospitalization for HF than patiromer [55]. Together, the DIAMOND and REALIZE-K trials show that potassium binders allow MRA and RAAS inhibitor doses to be maintained. However, the maintained dose has yet to be shown to translate into fewer HF events. Furthermore, given the direction of the exploratory signal, close attention should be paid to volume status and the calcium-based binder should be favored for patients whose congestion is difficult to control.

Finerenone is a non-steroidal, selective MRA with a differing tissue distribution compared to spironolactone. It has been evaluated in three large-scale trials covering the cardiorenal spectrum. In the FIDELIO-DKD trial, which included 5734 participants with type 2 diabetes, an eGFR between 25 and 75 mL/min/1.73 m^2^, and significant proteinuria, finerenone reduced the primary composite outcome of kidney failure, sustained decline in eGFR of at least 40% from baseline, or renal death (HR 0.82, 95% CI 0.73–0.93, *p* = 0.001). A 14% relative reduction was observed in the cardiovascular composite (cardiovascular death, non-fatal myocardial infarction, non-fatal stroke, or hospitalization for HF:HR 0.86, 95% CI 0.75–0.99, *p* = 0.03) [56]. The FIGARO-DKD trial extended the population to participants with less advanced kidney disease at baseline and reported a 13% reduction in the cardiovascular composite (HR 0.87, 95% CI 0.76–0.98, *p* = 0.03) [57]. The FINEARTS-HF trial (*n* = 6001) evaluated finerenone in HFmrEF and HFpEF (LVEF ≥ 40%) and showed a significant reduction in the composite of cardiovascular death and total heart failure exacerbations (rate ratio 0.84, 95% CI 0.74–0.95, *p* = 0.007) over a median follow-up of 32 months. The benefit was observed in patients receiving and not receiving SGLT2 inhibitor therapy at baseline [58]. The FINE-HEART pre-specified pooled analysis, including 18,991 participants from the FIDELIO-DKD, FIGARO-DKD, and FINEARTS-HF trials, reported a nominal reduction in all-cause mortality (HR 0.91, 95% CI 0.84–0.99, *p* = 0.027), a lower rate of heart failure hospitalizations (HR 0.83, 95% CI 0.75–0.92, *p* < 0.001), and a decrease in the composite kidney outcome (HR 0.80, 95% CI 0.72–0.90, *p* < 0.001). Cardiovascular death was not significantly reduced, although treatment effects on mortality and heart failure events remained consistent in patients receiving concomitant SGLT2 inhibitors or glucagon-like peptide-1 (GLP-1) receptor agonists [59]. Mechanistically, finerenone and SGLT2 inhibitors operate through complementary pathways, and subgroup analysis from the FINE-HEART trial supports the additive interpretation [59]. The phase-2 CONFIDENCE trial subsequently showed that simultaneous initiation of finerenone and empagliflozin produced an additive reduction in urinary albumin-to-creatinine ratio at six months relative to either agent alone in patients with CKD and type 2 diabetes [60]. An outcome trial powered for cardiovascular or kidney composite endpoints has not yet been reported.

## 6. SGLT2 Inhibitors Across the Ejection Fraction Spectrum in CKD

The pharmacological evidence reviewed thus far covers GDMT in HFrEF and finerenone across the LVEF range, mirroring both the historical trajectory of clinical trials and the design of the ADQI/AHA framework. Yet a substantial proportion of patients with type 2 or type 4 CRS present with HF with preserved or mildly reduced ejection fraction, and the SGLT2 inhibitor evidence in this population deserves separate treatment. Many patients with CKD who develop symptomatic or pre-symptomatic HF show preserved or borderline systolic function that becomes apparent only through echocardiographic strain analysis or serial NT-proBNP monitoring before overt decompensation.

The EMPEROR-Preserved trial demonstrated that empagliflozin reduces the composite of cardiovascular death and heart failure hospitalization in patients with LVEF > 40% [61], and the DELIVER trial reproduced the signal for dapagliflozin across an LVEF spectrum extending down to 40%, with worsening HF (defined as hospitalization or urgent visit) included in the primary composite alongside cardiovascular death [62]. Pre-specified analyses of patients with coexisting CKD confirmed preserved efficacy in both trials, although the renal endpoints were less consistent than in HFrEF populations or in the dedicated kidney outcome DAPA-CKD and EMPA-KIDNEY trials. The relative risk reductions in HFpEF appear smaller than those documented in HFrEF, but the absolute benefit scales with baseline risk, which CKD amplifies considerably. These results establish an effective pharmacological option in HFpEF, where none previously existed, and make the patient with preserved ejection fraction and CKD a candidate for combined SGLT2 inhibition and finerenone, building on the FINEARTS-HF and FINE-HEART data presented in Section 5.

A management framework dedicated specifically to the HFpEF–CKD overlap has yet to emerge from prospective trials enrolling this phenotype as the primary target population. Most available evidence derives from subgroup analyses, and the question of how to sequence or combine SGLT2 inhibitors and finerenone in CKD patients with preserved ejection fraction remains open. That uncertainty concerns the order of agents and leaves the indication itself intact; it should not be used as a reason to defer SGLT2 inhibition in HFpEF with CKD.

## 7. Glucagon-like Peptide-1 Receptor Agonists in the Cardiorenal Patient

The case for GLP-1 receptor agonist therapy in cardiorenal disease rested for years on cardiovascular outcomes from trials in type 2 diabetes whose kidney endpoints were exploratory. The FLOW trial addressed this gap directly. Perkovic and colleagues randomized 3533 patients with type 2 diabetes and CKD to once-weekly subcutaneous semaglutide 1.0 mg or placebo, with every participant on background RAAS inhibition. The primary composite endpoint—comprising kidney failure, a sustained ≥50% decline in eGFR, or death from kidney or cardiovascular causes—occurred in 331 of 1767 semaglutide-treated participants compared to 410 of 1766 on placebo. This 24% relative risk reduction (HR 0.76, 95% CI 0.66–0.88, *p* = 0.0003) prompted early termination at the pre-specified interim analysis [63]. The kidney-specific composite tracked the primary result (HR 0.79, 95% CI 0.66–0.94), and cardiovascular death fell by 29% (HR 0.71, 95% CI 0.56–0.89). Hierarchical testing returned an 18% reduction in major adverse cardiovascular events (HR 0.82, 95% CI 0.68–0.98, *p* = 0.029) and a 20% reduction in death from any cause. The mean annual eGFR slope separated the two arms by 1.16 mL/min/1.73 m^2^ (95% CI 0.86–1.47, *p* < 0.001) in favor of semaglutide. Sustained over years, that gap translates into a meaningful delay before patients with cardiorenal disease reach kidney failure. Eligibility was tied to two strata of disease severity: an eGFR of 50–75 mL/min/1.73 m^2^ with UACR 300–5000 mg/g, or an eGFR of 25 to <50 mL/min/1.73 m^2^ with UACR 100–5000 mg/g. The cohort’s mean baseline eGFR was 47 mL/min/1.73 m^2^, with roughly 80% of participants enrolling below 60 mL/min/1.73 m^2^ and about two-thirds carrying macroalbuminuria. The benefit on the primary outcome held across baseline eGFR strata, including patients below 30 mL/min/1.73 m^2^, and across UACR strata up to ≥2000 mg/g.

Heart failure outcomes were examined separately. Pratley and colleagues examined a composite of heart failure events (hospitalizations and urgent visits) plus cardiovascular death, with adjustment for baseline cardiovascular history. Semaglutide reduced the composite, and no heterogeneity emerged across prior heart failure history, NYHA class, or LVEF category. Baseline event risk was concentrated in NYHA class III and in the HFrEF subset. Given the smaller size of that subset, the stratum-level estimate warrants caution [64]. Together with Kosiborod and colleagues’ STEP-HFpEF program, which documented symptomatic and functional benefit from semaglutide in obesity-related HFpEF [65], the FLOW findings suggest a role for GLP-1 receptor agonist therapy as an adjunct to RAAS inhibition, SGLT2 inhibitors, and non-steroidal MRAs in type 2 diabetes with CKD, pending formal incorporation into clinical guidelines. Extension to non-diabetic CKD or to advanced kidney failure has not yet been supported by adequately powered randomized data. The FLOW trial tested semaglutide alone, and its kidney results cannot be extended to the class as a whole. Tirzepatide, a dual GIP/GLP-1 receptor agonist, lowered cardiovascular death or worsening HF in obesity-related HFpEF in the SUMMIT trial [66], and a dedicated kidney outcome trial of tirzepatide has yet to report. The KDIGO 2026 report addresses GLP-1 receptor agonists as emerging agents with cardiovascular and kidney benefit in diabetic CKD, while noting that evidence in advanced CKD remains limited [2]. The sequencing of these agents remains an unresolved practical question. In a diabetic cardiorenal patient initiating therapy from a low pharmacological baseline, the order in which the disease-modifying classes are introduced and the interval before finerenone is added to an SGLT2 inhibitor are decisions taken on pragmatic grounds, since trial evidence on sequence is lacking. While the mechanistic rationale for combination therapy is strong, the trial-level case for any specific sequence has not been made.

The FLOW population was defined by diabetic kidney disease. Consequently, the application of these findings to non-diabetic CKD relies on mechanistic plausibility and emerging data more than on randomized confirmation. The eGFR slope finding has implications that reach beyond the cardiorenal context. A between-arm separation of 1.16 mL/min/1.73 m^2^/year sits in the same broad range as the slope differences from the DAPA-CKD and EMPA-KIDNEY trials, although differences in slope analysis methodology preclude any tighter comparison. Although no dedicated factorial or head-to-head trial appears to have tested the additivity of GLP-1 receptor agonist therapy and SGLT2 inhibition when the two are deployed together, the FINE-HEART pooled analysis offers indirect reassurance, with finerenone treatment effects preserved in patients concurrently exposed to either class [59]. The question for the next generation of pragmatic trials is how the three classes function together over a multi-year horizon. In practice, the sequencing uncertainty turns into inertia when the absence of a trial-defined order is used to postpone the third or fourth agent altogether. The RENEW-CKD figures in Section 8.1 illustrate the result.

## 8. Therapeutic Inertia in Cardiorenal Syndrome

The determinants of delayed treatment intensification can be grouped at the patient, clinician, and system levels.

### 8.1. Mapping Inertia in the Cardiorenal Continuum

Phillips defined therapeutic inertia as the clinician’s failure to initiate or intensify treatment when the indication is unambiguous [15]. Khunti and Davies broadened this notion to clinical inertia, encompassing delayed screening, missed referrals, overdue treatment review, and reluctance to deprescribe medication that is no longer needed [16]. Both formulations are directly relevant to cardiorenal practice, where every prescribing decision carries renal function implications routinely interpreted as contraindications by conservative clinicians. Usherwood subsequently formalized the contributory mechanisms into clinician-side and patient-side determinants. On the clinician side, these include knowledge gaps, discomfort with diagnostic or therapeutic uncertainty, safety concerns over treatment intensification, and limited consultation time. On the patient side, prominent factors include older age, reduced life expectancy, multimorbidity, and parameters already close to target [67]. Failures of clinical action take several forms in routine practice: an echocardiogram never ordered, an NT-proBNP never measured, RAAS inhibitors stopped over a trivial creatinine rise, or sub-target discharge doses without a titration plan. The third meets the strict definition of therapeutic inertia, while the others fall under the broader umbrella of clinical inertia.

The clearest quantification of the cost of delayed intensification comes from glycemic management in type 2 diabetes, a population that differs from the cardiorenal one, so the figures that follow are offered as an analogy and carry less weight than the HF-specific registry data that come after them. In a UK Clinical Practice Research Datalink cohort of 105,477 patients with newly diagnosed type 2 diabetes followed for a median of 5.3 years, Paul and colleagues compared two groups: patients with HbA1c persistently ≥7% who intensified after >12 months, and patients intensified within 12 months at HbA1c < 7%. Delayed intensification was associated with adjusted hazard increases of 67% for myocardial infarction, 64% for HF, 51% for stroke, and 62% for the composite cardiovascular endpoint, with comparable findings at the 7.5% HbA1c threshold. The observational design supports association and cannot establish causation [68]. In HF, the CHAMP-HF benchmark from Section 1 quantifies the prescribing shortfall, with CKD independently worsening initiation and dose attainment across most classes [14]. The EVOLUTION-HF trial mapped GDMT patterns in routine-care databases from Japan, Sweden, and the United States during the 12 months after hospitalization for HF. At one year, target-dose attainment was 75.7% for dapagliflozin, 28.2% for sacubitril/valsartan, 20.1% for ACE inhibitors, 6.7% for ARBs, 7.2% for β-blockers, and 5.1% for MRAs. The two novel agents (dapagliflozin and sacubitril/valsartan) were initiated later than the four other classes across all three healthcare systems [69]. One structural feature of this comparison limits its interpretation: dapagliflozin is prescribed at a single 10 mg dose, so target-dose attainment coincides with initiation, whereas the other classes reach target only after several up-titration steps. The figure for dapagliflozin records how often the drug was initiated and says little about titration quality relative to the other classes. A pan-European panel statement coordinated by Girerd and colleagues identified physicians’ concerns over renal function and electrolytes as the principal driver of suboptimal dosing [70]. In the EPIC-HF cohort, the “clinical inertia” category in the non-intensification taxonomy accounted for 22.7–31.6% of decisions across the four GDMT classes, with parallel ranges of 18.2–31.6% for heightened concern about adverse effects and 15.6–31.8% for prioritization of competing problems [71].

The HFA scientific statement on therapeutic inertia in HFrEF maps organizational and educational barriers to GDMT delivery within that single phenotype [72]. In CRS, the same barriers operate, with the additional friction that every prescribing decision must be defended in two organ systems simultaneously. Gembillo and colleagues proposed a three-level taxonomy for diabetic kidney disease, allocating the determinants of delayed intensification to patient, clinician, and system levels. This framework transfers to CRS [17] and structures the next three subsections. Types 1 and 2 CRS, clinical inertia manifests as reluctance to maintain GDMT through hemodynamic eGFR changes. Type 4 fails earlier in the care cascade during surveillance: the clinical archetype is the patient with CKD presenting to cardiology or nephrology in whom left ventricular hypertrophy and subclinical diastolic dysfunction are detected only on a delayed first echocardiogram or NT-proBNP measurement. HFrEF-focused implementation guidance does not capture this trajectory.

Cardiorenal cohort data confirm that the inertia long documented in HFrEF registries persists when kidney dysfunction is present. The Hellenic Cardiorenal Morbidity Snapshots (HECMOS 1 and 2) comprised two cross-sectional surveys of cardiology inpatients across Greece, conducted on 3 March 2022 (55 departments, n = 923) and on 5 June 2024 (71 departments, n = 1222). SGLT2 inhibitor use among patients with CKD doubled between the two waves, from 15% to 30.4%. Finerenone reached only 1.7% of eligible patients in the 2024 survey, and RAAS inhibitor uptake stayed essentially flat (41.7% in 2022 vs. 42.1% in 2024) [73]. The two-year improvement is real but partial: most eligible patients still leave the hospital without the classes with the strongest cardiorenal evidence base. In a call-to-action narrative review built on the KDIGO 2024 CKD guideline, Wanner and colleagues described a consistent pattern of gaps between recommendation and practice: suboptimal RAAS inhibitor dosing despite proven cardiorenal benefit, low SGLT2 inhibitor uptake among patients with CKD, and low or transient use of potassium binders, which constrains RAAS inhibitor titration after an episode of hyperkalemia [74]. The same pattern recurs in nephrology and in primary care, irrespective of the health system examined, and it is documented across multinational diabetology surveys [75]. RENEW-CKD, a contemporary multicenter prospective Italian analysis, followed 4523 patients with non-dialysis CKD (stage 3–5, or stage 1–2 with UACR > 30 mg/g) across 30 nephrology clinics over two visits six months apart. At six months, therapeutic inertia rates for the four evidence-based anti-albuminuric classes were 85% for RAAS inhibitors and 90% for SGLT2 inhibitors overall (92% in non-diabetic and 85% in diabetic patients). Among patients with type 2 diabetes, inertia reached 92% for GLP-1 receptor agonists and 96% for finerenone. Independent predictors of non-prescription were class-specific: for RAAS inhibitors, these included low-normal blood pressure and an eGFR below 30 mL/min/1.73 m^2^; for SGLT2 inhibitors, these included older age, an eGFR below 45 mL/min/1.73 m^2^, the absence of diabetes or cardiovascular disease, and a low-normal UACR [76]. Even nephrology outpatient services, where these classes might be expected to enter the regimen most reliably, showed the highest inertia rates in patients with the greatest baseline absolute risk.

### 8.2. Patient-Level Barriers

Patients with concurrent heart and kidney disease typically face a higher comorbidity and polypharmacy burden than those with either condition alone. Maintaining the four pillars of cardiorenal pharmacology imposes a substantial cognitive load on patients already affected by fatigue and reduced executive function. Depression is more common in this population; the figures available come from diabetic kidney disease, where prevalence reached 17.8% in patients with diabetes compared to 9.8% in non-diabetic controls [17]. They are cited here by extrapolation since comparable estimates from cardiorenal cohorts are scarce. Clinicians too often label depressive disengagement as non-compliance. Fear of dialysis is common in nephrology outpatient encounters: some patients with CKD may refuse RAAS inhibitors or SGLT2 inhibitors, believing that an eGFR dip on starting the drug means accelerated progression to renal replacement. Cost is a persistent constraint: even where SGLT2 inhibitors and finerenone are approved and reimbursed, residual co-payment is enough to drive early discontinuation in lower-income patients. Anxiety about injection-related adverse effects continues to slow GLP-1 receptor agonist uptake in cardiorenal practice [64]. Visual and motor impairments common in older patients further compromise the practical handling of multi-drug regimens, and initial denial after a CKD or heart failure diagnosis can postpone the start of disease-modifying therapy.

### 8.3. Clinician-Level Barriers

The barrier most often cited by prescribers in the HF survey literature caution about changes in creatinine. In a pan-European panel statement, Girerd and colleagues identified concerns about renal function and electrolytes as the principal driver of GDMT underdosing below target [70]. The HFA statement on the renal effects of GDMT [44] established that early hemodynamic eGFR drops following initiation or up-titration of GDMT do not predict adverse outcomes. Chatur’s pooled analysis of the run-in periods of the PARADIGM-HF and PARAGON-HF trials [41] confirmed this pattern specifically for sacubitril/valsartan, with no erosion of long-term treatment benefit. The behavior persists despite this evidence: pre-emptive dose reduction is triggered by laboratory shifts that, on current evidence, reflect hemodynamic nephroprotection and give no indication of drug toxicity.

Organizational layout compounds the problem. SGLT2 inhibitor initiation at an eGFR of 25 mL/min/1.73 m^2^ is deferred from cardiology to nephrology on the assumption that the relevant expertise rests there, while β-blocker or ARNi up-titration is referred back to cardiology by the nephrologist who received the patient. The combined cardiorenal-prescribing problem ends without an owner, and the patient is discharged or seen in follow-up without an identified prescriber for the underused classes. This description rests on clinical observation and is offered as informed commentary: the frequency of such unowned handovers has yet to be measured in a cardiorenal cohort. A position statement co-authored by diabetologists from 15 European and Mediterranean countries reported that patients with type 2 diabetes at high cardiovascular risk who also carry CKD or HF remain more likely to receive DPP-4 inhibitors than either SGLT2 inhibitors or GLP-1 receptor agonists, with no contraindication explaining this pattern. This preference is attributable to prescribing habit, since no pharmacological reasoning accounts for it [75]. Clinicians trained before these classes entered routine use remain reluctant to start SGLT2 inhibitors and finerenone, and brief outpatient encounters leave little room for the shared decision-making that initiating a new cardiorenal agent demands.

Advanced CKD carries the highest absolute event rate but receives the lowest treatment intensification rates. In the DAPA-CKD trial, the placebo-arm rate of the composite primary endpoint (sustained ≥50% eGFR decline, end-stage kidney disease, or kidney/cardiovascular death) was 14.9 per 100 patient-years among patients with an eGFR below 30 mL/min/1.73 m^2^, which was nearly triple the 5.1 per 100 patient-years observed when the eGFR exceeded 45 [42]. In a meta-analysis of reconstructed time-to-event data from five SGLT2-inhibitor heart-failure trials (n = 21,947), Chen and colleagues identified the first nominal statistically significant reduction in worsening HF or cardiovascular death at day 26, with sustained statistical significance from day 118 (3.93 months) onward [77]. For patients with type 2 or type 4 CRS whose composite event rate approaches 15 per 100 patient-years, early initiation translates into a substantial absolute risk reduction.

### 8.4. System-Level Barriers

Cardiorenal-prescribing responsibility is divided across cardiology, nephrology, internal medicine, and primary care, with no nominated lead in most institutions. Beyond clinician-level fragmentation, structural deficiencies that no individual clinician can correct compound the problem. Hospital electronic health record (EHR) decision-support systems frequently treat HF and CKD as separate problems, and their eGFR alerts do not distinguish a functional hemodynamic dip from true kidney injury. Formulary restrictions on SGLT2 inhibitors and finerenone in some settings still apply eGFR thresholds that more recent trial data have superseded. Post-discharge up-titration pathways are a particular weakness. During the first 90 days after an acute heart failure admission, when rehospitalization risk is highest (peaking in the first 30 days) and the marginal benefit of intensification is greatest, management is typically left to outpatient discretion. The STRONG-HF trial randomized 1078 patients hospitalized for acute HF to usual care or to a high-intensity protocol that titrated RAAS blockade, β-blockers, and MRAs to full recommended doses within two weeks of discharge, with NT-proBNP measurements at one, two, three, and six weeks. The primary composite of all-cause death or heart failure readmission within 180 days fell from 23.3% to 15.2% in the intervention arm (adjusted risk difference 8.1 percentage points; adjusted risk ratio 0.66, 95% CI 0.50–0.86, *p* = 0.0021) [78].

Administrative pressures compound the clinical challenge. Formulary switching, prior-authorization delays, pharmacy-driven generic interchange, and substitution of cardiorenal medications with cheaper alternatives generate repeated prescription changes that disrupt patient adherence and the prescribing confidence needed to keep a regimen stable. These observations derive from clinical practice. Because no cardiorenal cohort appears to have quantified them, this paragraph should be interpreted as informed commentary.

Without shared data, large-scale institutional comparison of cardiorenal prescribing is impractical. Health technology assessments routinely omit readmission costs from CRS cost-effectiveness models and so understate the savings achievable through intensified pathways that would, if modeled, fall well below conventional incremental cost-effectiveness ratio (ICER) thresholds.

### 8.5. Strategies to Overcome Cardiorenal Inertia

Implementation evidence from diabetic kidney disease is partly transferable to cardiorenal practice [17]. Patient education, delivered through written materials and supported by digital and interactive formats, improves adherence and medication literacy. Risk-factor modification covers preset meal planning, home blood pressure monitoring with remote data transmission, alcohol-reduction counseling, and supported smoking cessation pathways. Psychological assessment and management remain underused in CRS services, despite their role in addressing depressive symptoms that compromise adherence.

At the clinician level, the priority is to counter creatinine anxiety. The “treat-to-success” framing developed in diabetic kidney disease [17], which replaces the “treat-to-failure” habit, supports continued titration until target doses are reached or a contraindication is documented. Institutional training should incorporate the HFA consensus on the renal effects of GDMT [44], Chatur’s pooled analysis of the run-in periods of the PARADIGM-HF and PARAGON-HF trials [41], and the KDIGO 2024 eGFR change thresholds [10]. Multidisciplinary teams that bring cardiology and nephrology together with pharmacy, nursing, dietetics, and social work cover the breadth of expertise the syndrome requires [72]. Pharmacist-led medication reviews can identify drug interactions, renal function-based dose adjustments, duplications, contraindication patterns, and therapeutic-class omissions that a busy generalist may miss. Clinical audit cycles embedded into continuing professional development allow clinicians to compare their own prescribing against external reference points, while quantitative feedback drives behavioral change more reliably than exhortation.

The system-level interventions best supported by evidence are protocolized. STRONG-HF supplied the proof of concept for protocolized post-discharge up-titration [78]. CRS-specific EHR alerts, for instance when a RAAS inhibitor is stopped without a documented acute indication, when SGLT2 inhibitor eligibility goes unreviewed in a patient with HF and an eGFR of 20–45 mL/min/1.73 m^2^, when finerenone candidacy is missed in a patient with type 2 diabetes and persistent albuminuria, or when potassium-binder candidacy is overlooked after prior hyperkalemia, embed guidance into workflow steps. Telemedicine, piloted in cardiorenal and diabetic kidney disease programs with mixed results, remains a complement to in-person care until definitive trials clarify the appropriate balance of contact frequency and clinician workload. Linking existing national cardiac and renal registries would enable institutional benchmarking that is currently impractical. A formal cardiorenal counterpart to the American Diabetes Association Overcoming Therapeutic Inertia initiative, ideally co-developed by KDIGO, ERA, the European Society of Cardiology, and the HFA, would supply the educational and decision-support framework that most institutions still lack (Table 2).

## 9. Multidisciplinary Cardiorenal Care and Guideline Implementation

The distribution of prescribing responsibility across multiple specialties has real-world implications that a multidisciplinary framework is designed to address. The 2019 KDIGO Controversies Conference on Heart Failure in CKD proposes a framework that remains the most direct international consensus on the operational problem [9]. This principle was included in broader recommendations on team-based collaborative care in the 2024 KDIGO guideline on CKD [10]. Both the 2021 ESC HF guideline and its 2023 focused update assume integrated multidisciplinary delivery as a precondition, with explicit acknowledgement that supporting infrastructure varies considerably between systems [52,79]. The HFA consensus on the renal effects of GDMT includes all relevant drug classes and all electrolyte and eGFR trajectories and provides a prescribing reference for cardiorenal teams to standardize practice between systems [44].

A dedicated cardiorenal clinic could fill several gaps at once. Prescribing responsibility for the cardiorenal pillars is borne by a named clinician across visits. The same reasoning applies to finerenone eligibility in HFmrEF/HFpEF with concomitant CKD, to patiromer use as an enabler of continued MRA therapy after a hyperkalemia episode, and to initiation of SGLT2 inhibitors when the eGFR sits between 20 and 45 mL/min/1.73 m^2^.

Little implementation evidence specific to cardiorenal care exists, in contrast to HF alone. The ESC/HFA scientific statement on therapeutic inertia in HFrEF defines the diagnostic aspects of the problem within that single phenotype [72]. Section 8.1. discusses the HFrEF registry data that allow an estimate of the magnitude of the gap in patients with HF [14,69,71]. A similar pattern is described in outpatient nephrology by RENEW-CKD [76]. The STRONG-HF trial showed that a protocolized approach can decrease the inertia rate within a defined post-discharge window [78]. To date, no dedicated cardiorenal trial has been reported comparing standard integrated care to a defined cardiorenal pathway with a named prescriber and structured handover.

The personalized dimension complements the multidisciplinary one. Cardiorenal patients are extremely heterogeneous in terms of congestion phenotype, residual albuminuria, hemodynamic dependence on RAAS tone, potassium handling, frailty, cognitive function, and socioeconomic context. Each of these variables changes the risk–benefit ratio for individual drug classes at individual doses. The multimodal point-of-care imaging toolkit discussed in Section 2 provides phenotype-level information that is not captured by static laboratory values [18,22,25,26]. The longitudinal biomarker trajectories described in Section 3 add a molecular layer to the hemodynamic picture. Personalized nephrology, as developed in the broader precision-medicine literature [27], is characterized by matching the GDMT drug, sequence, dose, and monitoring intensity to the individual risk profile while staying within the guideline envelope. The STRONG-HF trial provides a working template: guided reassessment of NT-proBNP at weeks 1, 2, 3, and 6, with individualized titration limits [78]. The logical development is to expand this protocol to the cardiorenal population.

## 10. Economic Burden and the Cost Case for Acting Sooner

The cardiorenal population carries a very high financial cost, combining three of the most expensive chronic conditions on the current Medicare ledger: CKD, diabetes, and HF. According to the United States Renal Data System (USRDS), patients with all three diagnoses represent the most expensive subgroup in the registry. In 2016, the mean spending per patient per year was $39,506—more than double the $16,176 reported for CKD alone [6]. Hospitalization costs account for about 75–80% of heart failure costs. Kwok and colleagues reported a mean cost per admission of $13,807 among 2,645,336 patients in the Nationwide Readmission Database [80]. The total cost of HF in the US, including indirect costs, is projected to increase from about $31 billion in 2012 to $70 billion by 2030 [81]. Once a patient starts renal replacement therapy, Medicare spending on dialysis alone ranges from $87,000 to $99,000 per patient per year, with a total annual amount over $114 billion [6].

Health economic estimates for the cardiorenal armamentarium are favorable. McEwan and colleagues modeled DAPA-CKD from a lifetime payer perspective across three European systems and reported incremental cost-effectiveness ratios (ICERs) for dapagliflozin versus placebo of $8280, $17,623, and $11,687 per quality-adjusted life-year (QALY) gained in the UK, Germany, and Spain, respectively. These figures are quoted as published; the authors converted their results from local currency to US dollars in the main text, with native-currency outcomes reported in the supplementary material. Cost-effectiveness was judged against country-specific willingness-to-pay thresholds—likewise converted—of about $27,500 per QALY for the UK and $35,500 per QALY for Germany and Spain, so that each comparison is judged against its own local benchmark [82]. Tisdale and colleagues applied a Markov model from a US healthcare-sector perspective to non-diabetic CKD and estimated an ICER of $60,000 per QALY gained. Dapagliflozin increased life expectancy by two years and lowered the proportion of patients progressing to end-stage kidney disease requiring renal replacement therapy from 17.4% to 11% [83]. In a secondary analysis of the DAPA-HF trial, Parizo et al. reported an ICER of $83,650 per QALY gained for dapagliflozin compared to standard therapy in HFrEF, ranked as an intermediate value on ACC/AHA benchmarks [84].

In each of these analyses, the drug classes most frequently withheld from eligible patients showed a cost per QALY below conventional willingness-to-pay thresholds.

The economic argument for structured up-titration parallels the clinical one. As an order-of-magnitude estimate, if the outcome reduction observed in the STRONG-HF trial were applied to the approximately 809,000 primary-diagnosis HF discharges, the savings during hospitalization would exceed the marginal expense of nurse-led titration clinics. Delaying progression to end-stage renal disease saves approximately $90,000 in annual Medicare expenditures per patient who would otherwise progress to end-stage renal disease [6]. There is also a patient-related dimension that is missing in traditional cost models. Responder analyses of the 23-item KCCQ in the DAPA-HF trial showed that more patients on dapagliflozin than placebo achieved a clinically meaningful improvement in symptom burden, physical limitation scores, and global summary at 8 months [85].

## 11. Additional Considerations in Older and Frail Patients

The median real-world age of patients with HF and co-existing CKD is >75 years, and frailty is present in approximately 40% of HF outpatients, rising to almost 50% when the eGFR is <30 mL/min/1.73 m^2^ [86,87]. A prospective cohort study from Hospital Clínic de Barcelona showed that frailty was present in 39.9% of HF outpatients and increased gradually with worsening renal function, from 14% in those with an eGFR > 60 to 48% in those with an eGFR < 30 mL/min/1.73 m^2^. The combination of advanced CKD and frailty identified a subgroup with an adjusted hazard ratio of 5.02 (95% CI 2.46–10.22) for death or HF hospitalization, which was much higher than the risk associated with either condition alone [87]. In a similar Spanish multicenter study, CKD was validated as a marker of worse prognosis in older patients with chronic HF, but not as an independent predictor of one-year mortality in the presence of frailty. These results are in line with the interpretation that non-cardiac and non-renal competing risks become more dominant beyond a threshold of frailty severity, and prognosis is then driven more by the frailty phenotype than by organ-specific dysfunction [88]. Frail patients with HF and CKD have worse treatment outcomes than non-frail patients, especially in terms of hospitalization and mortality rates. Multi-drug regimens raise the risk of adverse interactions, reduced medication adherence, and extra strain on patients’ physical and cognitive resources [89]. This can have direct implication on therapeutic inertia, as frailty itself was independently associated with lower GDMT implementation. In a post hoc analysis of the GUIDE-IT trial by Khan and colleagues, participants with a high frailty burden were less likely to be started and up-titrated on β-blockers, RAAS inhibitors, and MRAs, and optimal triple therapy at study end was reached by 17.7% of frail participants compared to 28.4% of non-frail participants [90]. In frail patients, clinical inertia may be magnified, where patients are considered clinically stable despite a substantially increased risk of adverse outcomes [89]. Further evidence came from the EMPA-KIDNEY trial, where 76% of participants receiving five or more concomitant medications at randomization were characterized by substantial overlap of frailty, multimorbidity, and polypharmacy [91]. In the AGING-HF study, an observational cohort of geriatric patients with HF, SGLT2 inhibitor use was associated with lower all-cause mortality and fewer HF hospitalizations at one year, without an excess of serious adverse events [92]. The design leaves room for confounding by indication: frail patients judged fit to receive the drug are likely to have been healthier than those who were not offered it, so this association carries less weight than the randomized frailty subgroup analyses of the DAPA-HF and DELIVER trials discussed below. A 2026 meta-analysis of 20,844 older patients with HF also showed consistent reductions in all-cause mortality and cardiovascular death across strata of age 65–74, 75 years or older, diabetes status, kidney function, and SGLT2 inhibitor subtype with a particularly pronounced 40% relative reduction in HF rehospitalizations [93]. What frailty asks for is a re-calibration of goals. To do so responsibly, clinicians must first confront a fundamental shortcoming of the evidence base. Systematic under-representation of frail older patients exists in key trials in cardiorenal medicine. The mean age of the participants was 66.2 years in the DAPA-HF trial and 67.2 years in the EMPEROR-Reduced trial. Real-world eligibility analysis found that real-world eligible patients were on average 79–80 years old, had worse renal function, higher rates of atrial fibrillation, and substantially lower rates of diabetes than trial participants [94]. In the DAPA-HF, EMPEROR-Reduced, EMPEROR-Preserved, and DAPA-CKD trials, frailty was not formally assessed; only 16% of participants were aged ≥75 years in the STRONG-HF trial [95]. Patients with an eGFR < 20 mL/min/1.73 m^2^ are systematically excluded from clinical trials, despite representing a significant and growing proportion of the real-world cardiorenal population [96]. This systematic exclusion results in a compounded evidence gap. The patients most likely to be seen in cardiorenal clinics are old, frail, multimorbid, on polypharmacy, with advanced CKD, and they are precisely the patients for whom the strongest therapeutic recommendations are based on the weakest direct evidence. Frailty-stratified post hoc analyses offer reassurance without closing the gap. In the DAPA-HF trial, dapagliflozin reduced the risk of worsening HF or cardiovascular death across the 3 frailty index classes with a greater absolute reduction in event rate in the frailer patients (−7.9 events per 100 person-years in FI class 3 vs. −3.5 in FI class 1). Serious adverse events and drug discontinuation did not occur more often with dapagliflozin versus placebo regardless of the frailty class [97]. The prespecified frailty analysis of the DELIVER trial confirmed these findings in HFpEF and HFmrEF, again showing consistent relative risk reduction and numerically increased absolute benefit in the frailest tertile, findings consistent with the DAPA-HF trial and sacubitril/valsartan treatment across ejection fraction subtypes [98]. The post hoc analysis of the EMPA-KIDNEY trial from the CKD side showed empagliflozin to be safe, tolerated, and effective in reducing the risk of CRS and hospitalization in those with frailty, multimorbidity, and polypharmacy characteristics [91]. In the CREDENCE, DAPA-CKD, and EMPA-KIDNEY trials, there was no significant difference in nephroprotective effects in the age-stratified analyses compared to younger patients; hazard ratios for kidney endpoints were similar in age subgroups [99]. The clinical message is that frailty should not be a reason to deny SGLT2 inhibitors or other evidence-based cardiorenal therapies when indicated because the patients with the most to gain are often the frailest. Frailty changes the framework for monitoring, titration, and goal setting.

Frailty is common in older patients with HF, but patients with frailty and particularly those with severe frailty, cognitive impairment, or significant multimorbidity are underrepresented in HF trials. Recent pharmacological advances have reduced HF-related events and led to improved symptoms, but the benefit-to-harm ratio in this population needs to be assessed on a person-centered basis, with intensification decided on individual goals [95]. A cardiogeriatric approach encourages the formulation of tailored treatment plans, including deprescribing when drugs are not in line with the patient’s goals or are responsible for adverse effects. Randomized data suggest that stopping preventive medications in patients with limited life expectancy does not lead to worse outcomes and may improve quality of life [100]. Given the high prevalence of frailty and functional decline [100,101], early conversations about advanced care planning should be prioritized in elderly CRS patients. Careful multifactorial risk assessment, including a comprehensive geriatric assessment, should guide prescribing or deprescribing decisions for both diuretics and SGLT2 inhibitors in older HF patients at risk of volume depletion and falls [99]. Thus, frailty assessment using validated tools such as the Frailty Index, Clinical Frailty Scale, or Fried criteria should be incorporated as a standard component of cardiorenal clinic workflows.

## 12. Conclusions

The pharmacological landscape of CRS has been substantially redrawn by the trials of the past decade, and its diagnostic framework rests on an established international consensus [2]. The four pillars of HFrEF therapy (ARNi or RAAS inhibitors, β-blockers, MRAs, and SGLT2 inhibitors) retain their cardiovascular benefit across the CKD stages in which they have been tested, whereas the evidence for kidney protection differs by class: it is strongest for SGLT2 inhibitors, which have dedicated kidney-outcome trials, rests on secondary analyses for ARNi and MRAs, and is lacking for β-blockers, whose indication in CRS remains cardiac. The current guideline corpus, comprising the KDIGO 2024 CKD guideline, the HFA consensus on the renal effects of GDMT, and the KDIGO 2026 Heart Failure and CKD Conference report, offers a prescribing framework that a multidisciplinary care model can operate. However, substantive evidence gaps remain for patients with an eGFR below 20 mL/min/1.73 m^2^, those on dialysis, and other dedicated populations.

Some limitations of the review itself qualify these conclusions. The literature was assembled as a narrative review, without a registered protocol, a systematic search, or predefined inclusion criteria, so relevant studies may have been missed and the selection reflects the judgement of the authors. No formal risk-of-bias assessment was applied to the trials cited, and their design, size, and background therapy vary considerably, from the 188-patient CARRESS-HF trial to the pooled FINE-HEART dataset of almost 19,000 participants. The eGFR and albuminuria thresholds used for inclusion also differ across the trials discussed together: the DAPA-CKD trial required albuminuria and an eGFR of 25 mL/min/1.73 m^2^ or above, the EMPA-KIDNEY trial enrolled patients down to 20 mL/min/1.73 m^2^ without an albuminuria requirement, and the HF trials applied renal limits of their own. Consequently, statements about CKD in this review pool populations that are only partly comparable. Observational and randomized evidence have been distinguished where the distinction changes the interpretation, but several of the implementation arguments in Section 8 and Section 9 rest on registry data and clinical experience, and they should be weighed accordingly.

The unresolved trial questions are real but do not, on their own, explain the size of the implementation gap. Registry data document the size of the gap and show that recorded contraindications account for a small share of it [14,76]. While the remaining causes are inferred from prescriber surveys, non-intensification taxonomies [70,71], and clinical experience, observational data of this kind cannot establish them with certainty. On that evidence, unwarranted hesitation unjustified by clinical trial data appears to be a major contributor, and it is the most tractable barrier to address. How consistently proven therapies are delivered therefore determines how much of their benefit patients actually receive. Progress in cardiorenal medicine now depends as much on reliable delivery of established treatments as on the discovery of new ones.

## Figures and Tables

**Table 1:** Cardiorenal syndrome subtypes: temporal sequence, therapeutic priorities, and potential consequences of delay. The subtype classification and the temporal sequence follow the ADQI consensus and the AHA scientific statement [11,12,13]; the therapeutic priorities reflect more recent trial evidence and guidance discussed in Section 4 to Section 7, and the consequences of delay are a synthesis by the authors.

Type	Temporal Sequence	Therapeutic Priorities	Potential Consequences of Delay
1	Acute cardiac to kidney	Aggressive decongestion, including proximal tubule sodium handling blockade (acetazolamide, SGLT2i); early GDMT restart	Diuresis is withheld at the first creatinine rise; congestion is misclassified as pre-renal; sustained venous hypertension drives tubular hypoxia beyond the reversible threshold; the absence of proximal tubule sodium blockade allows the braking phenomenon to persist
2	Chronic cardiac to kidney	Sustained GDMT, SGLT2i, non-steroidal MRA, ARNi	RAASi and SGLT2i are either down-titrated or stopped at the expected hemodynamic eGFR dip; renal trajectory proceeds unchecked; the gap between trial-proven benefit and actual prescribing widens with advancing CKD stage
3	Acute kidney to heart	AKI management; continuous cardiac monitoring; electrolyte correction	Cardiac consequences of AKI, including arrhythmia, myocardial depression, uremic pericarditis, and conduction disturbances mediated by hyperkalemia and metabolic acidosis, are managed as separate problems; the unified cardiorenal mechanism is neither recognized nor treated as such
4	Chronic kidney to heart	CKD cardiovascular surveillance with echocardiography and NT-proBNP monitoring; finerenone in type 2 diabetes with albuminuric CKD; SGLT2i	Over time, LVH and diastolic dysfunction can develop undetected without echocardiographic or natriuretic peptide monitoring; patients are exposed to major hemodynamic stressors with a hidden structural vulnerability accrued silently through cardiorenal interaction
5	Systemic bidirectional	Treat the primary systemic driver; resist compartmentalized organ management	Specialist teams address cardiac and renal manifestations independently; the primary driver (e.g., sepsis, cirrhosis, or vasculitis) often receives delayed or insufficiently aggressive treatment while organ-level care proceeds in parallel

AKI, acute kidney injury; ARNi, angiotensin receptor–neprilysin inhibitor; CKD, chronic kidney disease; GDMT, guideline-directed medical therapy; LVH, left ventricular hypertrophy; MRA, mineralocorticoid receptor antagonist; NT-proBNP, N-terminal pro-brain natriuretic peptide; RAASi, renin–angiotensin–aldosterone system inhibitor; SGLT2i, sodium-glucose cotransporter 2 inhibitor; eGFR is “estimated Glomerular Filtration Rate”.

**Table 2:** Barriers to optimal cardiorenal care and evidence-informed countermeasures across patient, clinician, and system levels.

Level	Barrier	Clinical Consequence in CRS	Evidence-Informed Countermeasure
Patient	Polypharmacy burden, adherence fatigue, depression, cost, fear of dialysis or injection therapy, denial phase after diagnosis	Selective non-adherence to cardiorenal pillars; refusal of RAASi or SGLT2i on misconception of hemodynamic eGFR dip; early discontinuation of GLP-1 receptor agonist	Structured multimedia education that addresses the hemodynamic dip specifically; psychological assessment integrated into the CRS workflow; cost-conscious prescribing with explicit discussion; caregiver involvement in medication management [17]
Clinician	Creatinine anxiety; misreading of functional eGFR decline as drug toxicity; specialty silos; unfamiliarity with the contemporary cardiorenal armamentarium (SGLT2 inhibitors, finerenone, GLP-1 RAs); time-limited encounters	RAASi and SGLT2i withheld or down-titrated at the first eGFR dip [39,41]; DPP-4 inhibitors preferred over SGLT2i/GLP-1 RA in high cardiovascular-risk type 2 diabetes [75]; no named prescriber across specialties; 67% of eligible HFrEF patients on no MRA despite <2% contraindication [14]	HFA consensus on renal effects of GDMT as institutional standard [44]; KDIGO 30% eGFR-change threshold for reassessment [10]; multidisciplinary cardiorenal team with named prescribing lead [72]; pharmacist-led medication reviews; embedded clinical audit
System	Absent cardiorenal pathway; generic EHR alerts; formulary restrictions on SGLT2i/finerenone at low eGFR; no protocolized post-discharge titration; fragmented registries; administrative formulary churn	Post-discharge rehospitalization window of 30–90 days left to outpatient discretion; EVOLUTION-HF documents that SGLT2i are initiated later than any other class [69]; CHAMP-HF shows 1% of eligible patients on target doses of all three GDMT classes [14]	STRONG-HF protocol targeted rapid up-titration toward guideline-recommended doses within 2 weeks; 180-day death or HF readmission reduced from 23.3% to 15.2% [78]; CRS-specific EHR alerts; cardiorenal registry linkage across cardiac and renal data; formal CRS counterpart to ADA OTI [17]

Barriers to the delivery of guideline-directed cardiorenal therapy, organized by the level at which they arise, together with their clinical consequences in cardiorenal syndrome and countermeasures supported by current evidence. Countermeasures followed by a reference are supported by the cited evidence; those without a reference (pharmacist-led medication reviews, embedded clinical audit, CRS-specific EHR alerts, cardiorenal registry linkage, caregiver involvement) are organizational proposals of the authors and have not been tested in cardiorenal cohorts. ADA OTI, American Diabetes Association Overcoming Therapeutic Inertia initiative; CRS, cardiorenal syndrome; DPP-4, dipeptidyl peptidase-4; EHR, electronic health record; GDMT, guideline-directed medical therapy; GLP-1 RA, glucagon-like peptide-1 receptor agonist; HFrEF, heart failure with reduced ejection fraction; MRA, mineralocorticoid receptor antagonist; RAASi, renin–angiotensin–aldosterone system inhibitor; SGLT2i, sodium-glucose cotransporter 2 inhibitor;eGFR, estimated glomerular filtration rate; HFA, Heart Failure Association; KDIGO, Kidney Disease: Improving Global Outcomes; EVOLUTION-HF, Utilization of Dapagliflozin and Other Guideline-Directed Medical Therapies in Heart Failure Patients: A Multinational Observational Study Based on Secondary Data; CHAMP-HF, Change the Management of Patients with Heart Failure; STRONG-HF, Safety, Tolerability and Efficacy of Rapid Optimization, Helped by NT-proBNP Testing, of Heart Failure Therapies.

## Data Availability

No new data were created or analyzed in this study. Data sharing is not applicable to this article.

## Abbreviations

The following abbreviations are used in this manuscript:

ACC	American College of Cardiology
ACEi	Angiotensin-converting enzyme inhibitor
ADA OTI	American Diabetes Association Overcoming Therapeutic Inertia initiative
ADQI	Acute Dialysis Quality Initiative
AHA	American Heart Association
AKI	Acute kidney injury
ARB	Angiotensin receptor blocker
ARNi	Angiotensin receptor–neprilysin inhibitor
CI	Confidence interval
CKD	Chronic kidney disease
CRS	Cardiorenal syndrome
CVP	Central venous pressure
DKD	Diabetic kidney disease
DPP-4	Dipeptidyl peptidase-4
eGFR	Estimated glomerular filtration rate
EHR	Electronic health record
ERA	European Renal Association
ESC	European Society of Cardiology
FI	Frailty index
GDMT	Guideline-directed medical therapy
GLP-1 RA	Glucagon-like peptide-1 receptor agonist
HbA1c	Glycated hemoglobin
HF	Heart failure
HFA	Heart Failure Association
HFmrEF	Heart failure with mildly reduced ejection fraction
HFpEF	Heart failure with preserved ejection fraction
HFrEF	Heart failure with reduced ejection fraction
HR	Hazard ratio
ICER	Incremental cost-effectiveness ratio
IL-33	Interleukin-33
KCCQ	Kansas City Cardiomyopathy Questionnaire
KDIGO	Kidney Disease: Improving Global Outcomes
KIM-1	kidney injury molecule-1
LVEF	Left ventricular ejection fraction
LVH	Left ventricular hypertrophy
MCS	Mechanical circulatory support
MRA	Mineralocorticoid receptor antagonist
NT-proBNP	N-terminal pro–B-type natriuretic peptide
NYHA	New York Heart Association
OR	Odds ratio
QALY	Quality-adjusted life-year
RAAS	Renin–angiotensin–aldosterone system
RAASi	Renin–angiotensin–aldosterone system inhibitor
SGLT2	Sodium-glucose cotransporter 2
SGLT2i	Sodium-glucose cotransporter 2 inhibitor
sST2	Soluble ST2
UACR	Urine albumin-to-creatinine ratio
US	United States
USRDS	United States Renal Data System
VExUS	Venous Excess Ultrasound
